# Point Cloud Instance Segmentation with Inaccurate Bounding-Box Annotations

**DOI:** 10.3390/s23042343

**Published:** 2023-02-20

**Authors:** Yinyin Peng, Hui Feng, Tao Chen, Bo Hu

**Affiliations:** 1 Department of Electronic Engineering, Fudan University, Shanghai 200433, China; 2 Yiwu Research Institute, Fudan University, Yiwu 322000, China

**Keywords:** point cloud instance segmentation, learning with noisy labels, weakly supervised learning, self-distillation

## Abstract

Most existing point cloud instance segmentation methods require accurate and dense point-level annotations, which are extremely laborious to collect. While incomplete and inexact supervision has been exploited to reduce labeling efforts, inaccurate supervision remains under-explored. This kind of supervision is almost inevitable in practice, especially in complex 3D point clouds, and it severely degrades the generalization performance of deep networks. To this end, we propose the first weakly supervised point cloud instance segmentation framework with inaccurate box-level labels. A novel self-distillation architecture is presented to boost the generalization ability while leveraging the cheap but noisy bounding-box annotations. Specifically, we employ consistency regularization to distill self-knowledge from data perturbation and historical predictions, which prevents the deep network from overfitting the noisy labels. Moreover, we progressively select reliable samples and correct their labels based on the historical consistency. Extensive experiments on the ScanNet-v2 dataset were used to validate the effectiveness and robustness of our method in dealing with inexact and inaccurate annotations.

## 1. Introduction

The rapid development of 3D sensors, such as LiDARs and RGB-D cameras, has brought about an increasing amount of 3D data, thus promoting a wide range of applications, including autonomous driving [1], robotics [2], and medical treatment [3]. With the benefit of rich geometric information and the challenge of intrinsic irregularity, more and more attention has been paid to deep learning on 3D point clouds [4,5,6,7].

**Figure 1:**
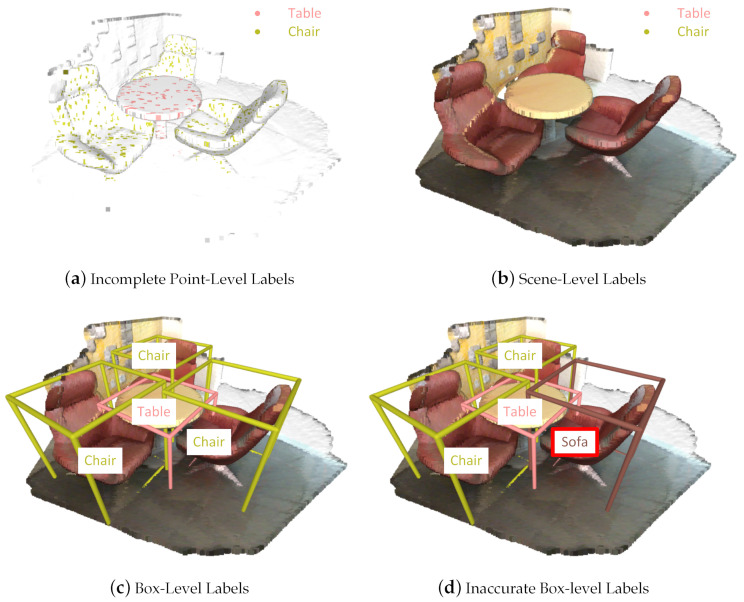
Illustration of various weak supervision methods for point cloud segmentation. (**a**) Incomplete point-level labels denote the classes to which a small fraction of points belong. (**b**) Scene-level (subcloud-level) labels indicate all of the classes appearing in the scene (subcloud). (**c**) Box-level labels indicate the class and location of each object. (**d**) Inaccurate box-level labels indicate the portion of boxes that are mislabeled. For example, a “chair” is mislabelled as a “sofa”.

As one of the fundamental tasks in 3D scene understanding, point cloud instance segmentation aims to predict the semantic label of each point and simultaneously distinguish points within the same class but in different instances. Numerous deep learning methods have been proposed to achieve progressively better performance [8,9,10,11,12,13,14]. However, the success of most existing segmentation methods depends heavily on accurately and densely annotated training data, which are time-consuming to collect. For example, it takes about 22.3 min to annotate all of the points of one scene in ScanNet [15]. To alleviate the point-level annotation burden of full supervision, a handful of methods have recently taken weak supervision into consideration; they mainly included incomplete [16,17,18] and inexact supervision [19,20,21,22]. The different kinds of weak supervision are illustrated in Figure 1.

For incomplete supervision, current research works perform semi-supervised learning through self-training, self-supervision, label propagation, etc. However, the way of choosing the small fraction of points to annotate is crucial for the segmentation performance. To represent the location of an instance, SegGroup [18] picks the largest segment, and CSC [23] finds exemplary points through active sampling. In other words, additional labeling efforts are required to implement point cloud instance segmentation with incomplete supervision.

For inexact supervision, there exist two leading types, i.e., scene-level (subcloud-level) and box-level supervision. Since it is difficult to extract object localization information from scene-level or subcloud-level tags [19], we focus on box-level supervision, which is of medium granularity and widely available. The key is to identify the foreground points in each bounding box without point-level instance labels. Box2Seg [21] uses attention map modulation and entropy minimization to generate pseudo-labels. SPIB [22] first conducts object detection with partial bounding-box labels and fulfills instance segmentation with three in-box refinement modules. Both of them need multi-stage training, and the box-level annotations are not fully exploited for instance segmentation. Box2Mask [20] allows each point to predict the box in which it belongs and trains the instance segmentation network from end to end with bounding-box annotations.

Nonetheless, the methods presented above implicitly assume that the labels are highly accurate, which may not be guaranteed in practice. Regardless of the granularity at which data are labeled, label noise exists due to the carelessness of annotators and the difficulty of annotating itself. When it comes to box-level label noise, Hu et al. [24] designed a noise-resistant focal loss for 2D object detection. With NLTE [25], it was found that it was essential for domain adaptive objective detection to address noisy box annotations, including miss-annotated boxes and class-corrupted ones. In 3D point cloud instance segmentation, the rough location information of most points is unaffected by slight fluctuations in box coordinates, while mislabeling the box semantics can lead to serious confusion of all of the in-box points. Therefore, we took the semantic label noise of each box into account while leaving the geometric coordinate noise for future work. The inaccurate box-level annotations are shown in Figure 1d. Since deep neural networks are highly capable of learning any complex function, it is easy to overfit inaccurate labels and reduce the generalization performance [26]. Thus, it is necessary to develop a noise-robust point cloud instance segmentation method. A recent work used PNAL [27] to study point noise in semantic segmentation, but it heavily relied on the early memorization effect, which increased the risk of discarding hard samples or those in the minor class. Furthermore, point cloud instance segmentation with inaccurate bounding-box annotations is even more challenging due to the granularity mismatch of given annotations and the target task. There is an urgent need to combat realistic label noise and sufficiently release the potential of box-level supervision in point cloud instance segmentation.

Extensive research has empirically demonstrated the success of knowledge distillation [28] in boosting the generalization ability, which is in great demand when learning with noisy labels. Traditional knowledge distillation transfers knowledge from a large teacher model, while self-distillation efficiently utilizes knowledge from itself and, thus, attracts more and more attention. As for theoretical analysis, there are various opinions that include label smoothing regularization [29], the multi-view hypothesis [30], and loss landscape flattening [31]. Similarly to our method, PS-KD [32] trained a model with soft targets, which were a weighted summation of the hard targets and the last-epoch predictions, and DLB [33] used predictions from the last iteration as soft targets. However, we considered the entire prediction history and maintained an exponential moving average of the predictions.

In this paper, we present a novel self-distillation framework based on perturbation and history (SDPH) to handle the challenge of point cloud instance segmentation with only inaccurate box annotations. Rather than distilling knowledge from a cumbersome teacher model or an extra clean dataset [34], we perform self-distillation by taking full advantage of self-supervision in the data and the learning process. To be specific, we assume that the predictions over the input point cloud are perturbation-invariant. Both geometric and semantic consistency regularization terms are included to provide additional supervision signals. Furthermore, by investigating the consistency of historical predictions, the model is able to locate and correct refurbishable samples with high precision. Finally, we apply temporal consistency regularization to fully utilize the history information and reduce the unstable prediction fluctuations that may hinder the label refurbishment. In a word, we utilize two kinds of consistency regularization to prevent the network from overfitting inaccurate labels and progressively correct the labels during the training process.

Overall, the main contributions of our paper are summarized as follows:

- To the best of our knowledge, this is the first work to simultaneously explore inexact and inaccurate annotations in the point cloud instance segmentation task.
- We propose a novel self-distillation framework for applying consistency regularization and label refurbishment by using data perturbation and history information.
- Extensive experiments were conducted to demonstrate the effectiveness of our method. The results on ScanNet-v2 show that our SDPH achieved comparable performance to that of densely and accurately supervised methods.

The rest of this paper is organized as follows. First, related research is described in Section 2. Next, we present our self-distillation framework in Section 3. Thereafter, the experimental results and analysis are provided in Section 4. Finally, Section 5 concludes the paper and points out future work.

## 2. Related Works

### 2.1. Point Cloud Instance Segmentation

Point cloud instance segmentation methods can be roughly divided into two categories: proposal-based methods and proposal-free methods.

#### 2.1.1. Proposal-Based Methods

Proposal-based methods first conduct object detection to generate region proposals and then perform binary classification to separate all of the foreground points in each proposal. GSPN [8] used an analysis-by-synthesis strategy to enforce geometric understanding in generating proposals with high objectness. These object proposals were further processed by Region-Based PointNet (R-PointNet) to obtain the final segmentation results. The method of 3D-SIS [35] first extracted 2D features from multi-view high-resolution RGB images and then projected them back to the associated 3D voxel grids. The geometry and color features were concatenated and fed into a fully convolutional 3D architecture. The method of 3D-BoNet [36] is a single-stage, anchor-free, and end-to-end trainable network. This method directly predicts a fixed number of bounding boxes and fuses the global information into a point mask prediction branch. The method of 3D-MPA [9] generates proposals through center voting, refines them by using a graph convolutional network, and obtains the final instances through proposal aggregation instead of non-maximum suppression.

#### 2.1.2. Proposal-Free Methods

Proposal-free methods focus on discriminative point feature learning and distinguish instances with the same semantic meaning through clustering. SGPN [11] first embeds all of the input points into feature space and then groups the points into instances based on the pairwise feature similarity, which is not scalable. JSIS3D [12] utilizes a multi-value conditional random field model to jointly optimize semantic labels and instance embeddings predicted by a multi-task point-wise network. ASIS [37] utilizes discriminative loss to pull embeddings of the same instance to its center and push those of different instances apart. Moreover, the association of instance segmentation and semantic segmentation further benefits each. PointGroup [38] predicts point offsets towards their respective instance centers and considers both the original point coordinates and the offset-shifted ones in the clustering stage. OccuSeg [13] introduces the occupancy signal to take part in multi-task learning and guide graph-based clustering. PE [14] encodes each point as a tri-variate normal distribution in the probabilistic embedding space, and a novel loss function that benefits both semantic segmentation and subsequent clustering was proposed. HAIS [10] performs point aggregation and set aggregation to progressively generate instance proposals. SoftGroup [39] groups points based on soft semantic scores to avoid error propagation and suppresses false positive instances by learning to categorize them as the background.

We follow the proposal-free approach because of its superior performance and flexible architecture. Nevertheless, we utilize inaccurate box-level supervision to learn point-level instance segmentation, which greatly alleviates the labeling cost.

### 2.2. Weakly Supervised Point Cloud Segmentation

Generally speaking, there are three typical types of weak supervision in machine learning: incomplete supervision, inexact supervision, and inaccurate supervision [40].

Most point cloud segmentation methods are concerned with incomplete supervision, where only a small subset of training data are given with labels [16,17,41,42,43]. This setting is also known as semi-supervised learning. Xu et al. [16] combined multi-instance learning, self-supervision, and smoothness constraints to achieve semantic segmentation with only 10 times fewer labels. Zhang et al. [41] constructed a self-supervised pre-training task through point cloud colorization and proposed an efficient sparse label propagation mechanism to improve the effectiveness of the weakly supervised semantic segmentation task. PSD [42] enforced the prediction consistency between the perturbed branch and the original branch, and a context-aware module for regularizing the affinity correlation of labeled points was presented. Liu et al. [17] adopted a self-training approach with a super-voxel graph propagation module. Similarly, SSPC-Net [43] built super-point graphs for dynamic label propagation and the coupled attention mechanism to extract discriminative contextual features.

Inexact supervision means that the training data are given with only coarse-grained labels, such as scene-level tags [19,44] and box-level annotations [20,21,22] in the segmentation context. MPRM [19] applied various attention mechanisms to acquire point class activation maps (PCAMs). After generating pseudo-point-level labels from PCAMs, a segmentation network could be trained in a fully supervised manner. WyPR [44] jointly performed semantic segmentation and object detection through a series of self- and cross-task consistency losses with multi-instance learning objectives. SPIB [22] first leveraged partially labeled bounding boxes to train a proposal generation network with perturbation consistency regularization and then predicted the instance mask inside each target box with three smoothness regularization and refinement modules. Box2Seg [21] learned pseudo-labels from bounding-box-level foreground annotations and subcloud-level background tags, and it achieved semantic segmentation through fully supervised retraining. Box2Mask [20] directly voted for bounding boxes and obtained instance masks via non-maximum clustering.

Inaccurate supervision means that the given labels are not always the ground truth. Although learning from noisy labels with deep neural networks has been explored very much, especially in image classification [45], few researchers have investigated noisy labels with increasing amounts of point cloud data. As the pioneering work in noise-robust point cloud semantic segmentation, PNAL [27] selected reliable points based on their consistency among historical predictions, and it corrected locally similar points with the most likely label, which was voted on in each cluster.

Most of the above weakly supervised methods focused merely on one type of weak supervision. However, the circumstances are usually more complicated in reality, where the label noise in particular is almost inevitable but often ignored. Thus, we consider both inexact and inaccurate supervision and develop a robust point cloud instance segmentation framework with inaccurate box annotations.

## 3. Our Method

### 3.1. Overview

**Figure 2:**
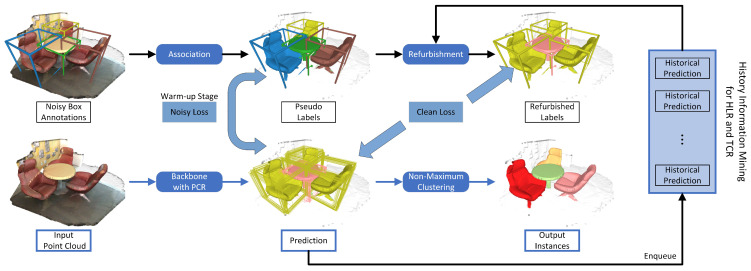
The training framework of self-distillation based on perturbation and history. We first generate pseudo-labels according to the point–box association (c.f. Section 3.2) and train a 3D sparse convolutional network with two types of consistency regularization, namely, PCR (c.f. Section 3.4.1) and TCR (c.f. Section 3.4.3). With the help of regularization, the model is able to perform label refurbishment (HLR, c.f. Section 3.4.2) with higher precision. Note that the noisy loss is used only in the warm-up stage, and afterward, it is replaced by the clean loss, since the cleaned (i.e., refurbished) labels are available.

The pipeline of our SDPH is depicted in Figure 2. Given a point cloud P with inaccurate bounding-box annotations, we first assign point-level pseudo-labels based on the spatial inclusion relations between points and boxes. This simple association process allows for a fully supervised training manner. After the label preparation, the backbone network takes a voxelized point cloud as input and produces embeddings for each voxel. To lessen the computational cost, we perform over-segmentation to group voxels into super-voxels. This basic training process will be introduced in Section 3.3. The final instances are obtained through super-voxel-level non-maximum clustering and backward projection.

Apart from the whole forward inference procedure, our self-distillation training framework consists of two main parts that leverage data perturbation and historical information. First, we construct a perturbed branch and keep the prediction consistency between the original branch and the perturbed one. Furthermore, the past predictions are fully exploited to select refurbishable samples and provide soft targets.

### 3.2. Pseudo-Label Generation

Since ground-truth point-level labels are not available, directly training a segmentation network with only box-level labels is infeasible. Therefore, we need to establish the box–point association first. Specifically, we categorize points according to the numbers of boxes containing them. If a point is contained in only one box, it is simply labeled as the unique box, which is represented by both the geometric coordinates and the semantic category. If a point is inside more than one box, the smallest one is associated with it. A point is treated as background if it is outside all of the boxes.

Let B denote a set of box annotations, with each box $b\in R^{7}$ representing its three-dimensional center, three-dimensional size, and one-dimensional semantic label. For clarity, we use $p_{i}\in b_{j}$ ($p_{i}\notin b_{j}$) to show that the *i*-th point is (not) contained by the *j*-th box. The pseudo-labels are generated through the following mapping function.

(1) $$\phi \left(p_{i}\right)=\left\{\begin{array}{cc} b_{j}, & j=\underset{j\in \{k|p_{i}\in b_{k}\}}{\arg\min}\mathrm{sizeof}\left(b_{j}\right),\\ \mathrm{background}, & \forall j,p_{i}\notin b_{j}. \end{array}\right.$$

Although this mapping function seems plausible, the generated point-level pseudo-labels inevitably suffer from inaccurate associations, as do the super-voxel-level pseudo-labels. The label quality will further degrade due to inaccurate box annotations, which motivated us to design a noise-robust self-distillation training framework.

### 3.3. Point Cloud Instance Segmentation Network

Before self-distillation, we introduce the basic point cloud instance segmentation network, where the labels are regarded noise-free. As a common choice, we adopted a UNet-like sparse convolutional network as the backbone [10,46,47]. The input point cloud is converted into volumetric grids and then fed into the backbone to extract voxel features, which are pooled into super-voxel features by using the over-segmentation results. Next, multiple output heads are applied to predict the semantic label, the associated box coordinates (offset and size), and the intersection-over-union (IoU) score of the predicted box with the ground-truth box. The basic network is trained with the following multi-task loss.

(2) $$L_{basic}=L_{sem}+L_{offset}+L_{size}+L_{score}.$$

Here, $L_{sem}$ is a normal cross-entropy loss for learning the semantics, which are formulated as

(3) $$L_{sem}=-\frac{1}{N}\sum_{i=1}^{N}\sum_{c=1}^{C}y_{ic}\log p_{ic},$$

where $y_{ic}$ represents the one-hot semantic label of the *i*-th super-voxel, $p_{ic}=\frac{\exp(z_{ic})}{\sum_{k=1}^{C}\exp\left(z_{ik}\right)}$ denotes the probability of being predicted as the *c*-th category, *N* is the number of super-voxels, and *C* represents the number of semantic categories. Note that the background is also included in the categories concerned with $L_{sem}$.

As for the box regression, we use the $L_{1}$ loss.

(4) $$\begin{array}{cc} L_{offset} & =\frac{1}{M}\sum_{i=1}^{M}{∥d_{i}-{\hat{d}}_{i}∥}_{1},\\ L_{size} & =\frac{1}{M}\sum_{i=1}^{M}{∥s_{i}-{\hat{s}}_{i}∥}_{1}, \end{array}$$

where *M* is the number of foreground super-voxels. $d_{i}$ and ${\hat{d}}_{i}$ represent the ground-truth and predicted offsets of the *i*-th super-voxel with respect to the associated box center, respectively. $s_{i}$ and ${\hat{s}}_{i}$ represent the corresponding box sizes.

To assist in the later non-maximum clustering and average precision calculation, the IoU score loss is defined as

(5) $$L_{score}=-\frac{1}{M}\sum_{i=1}^{M}\left[u_{i}\log v_{i}+\left(1-u_{i}\right)\log\left(1-v_{i}\right)\right],$$

where $u_{i}$ and $v_{i}$ represent the true and predicted IoUs between the predicted box and the associated ground-truth box, respectively.

At the inference stage, we follow Box2Mask [20] in performing non-maximum clustering (NMC), which follows exactly the same procedure of non-maximum suppression (NMS) in object detection. Instead of dropping redundant boxes, in NMC, they are collected to form clusters with the corresponding representative boxes. The semantic category of each cluster is assigned through a majority vote. Finally, the clustering structure of super-voxels is projected back to points, which completes the instance segmentation.

### 3.4. Self-Distillation Based on Perturbation and History

#### 3.4.1. Perturbation-Based Consistency Regularization

Since the original supervision method is inaccurate and untrustworthy, we turn to self-supervision, which has shown great power in deep learning. To provide additional supervision, we construct a perturbed branch and constrain the predictions of the perturbed and original branches to be consistent.

We adopt three kinds of perturbation strategies: scaling, flipping, and rotation. For scaling, we sample a scaling factor ξ from a uniform distribution U(0.8,1.2). The origin-centered scaling process is represented as $\tilde{P}=\xi \cdot P$, where $P\in R^{N_{p}\times 3}$ is the coordinate matrix of the input point cloud and $\tilde{P}$ is the transformed one. For flipping, we randomly sample the flipping indicators $f_{x},f_{y}$ from {−1,1}, where −1 means flipping over the corresponding axis. Thus, the flipping can be expressed as $\tilde{P}=P\cdot \mathrm{diag}\left(f_{x},f_{y},1\right)$. For rotation, the rotation angle θ around *z*-axis is denoted as $\theta_{z}$ and sampled from the uniform distribution U(0,2π). Rotating the point cloud means multiplying its coordinates with a rotation matrix as follows:

(6) $$\tilde{P}=P\cdot R\left(\theta {}_{z}\right)=P\cdot \left[\begin{array}{ccc} \cos\theta_{z} & \sin\theta_{z} & 0\\ -\sin\theta_{z} & \cos\theta_{z} & 0\\ 0 & 0 & 1 \end{array}\right].$$

**Figure 3:**
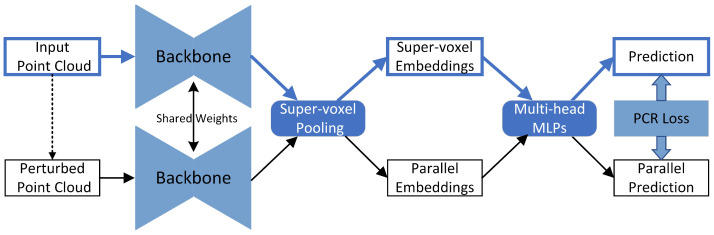
Illustration of the perturbation-based consistency regularization (PCR) module. We construct a parallel branch through data perturbation and force the output predictions of the two branches to be consistent. Note that the predictions include both semantics and geometry.

Obviously, both semantic and geometric predictions should be consistent between the two branches, i.e., the perturbation-based consistency regularization loss (“PCR loss” in Figure 3) is defined as

(7) $$L_{pcr}=L_{pcr}^{sem}+L_{pcr}^{geo}.$$

The KL-divergence and MSE losses are used as consistency regularization terms. To be specific, we formulate the semantic consistency loss as

(8) $$\begin{array}{cc} L_{pcr}^{sem} & =\frac{1}{N}\sum_{i=1}^{N}D_{KL}\left(p_{i}∥{\tilde{p}}_{i}\right)\\ \; & =\frac{1}{N}\sum_{i=1}^{N}\sum_{c=1}^{C}p_{ic}\log\frac{p_{ic}}{{\tilde{p}}_{ic}}. \end{array}$$

The geometric consistency loss is defined as

(9) $$L_{pcr}^{geo}=\frac{1}{N}\sum_{i=1}^{N}\left[∥{\tilde{\hat{o}}}_{i}-{\hat{\tilde{o}}}_{i}{∥}_{2}^{2}+{∥{\tilde{\hat{s}}}_{i}-{\hat{\tilde{s}}}_{i}∥}_{2}^{2}\right],$$

where $o_{i}$ represents the center of the *i*-th super-voxel’s associated box. In addition, $\hat{\cdot }$ indicates the predicted value, and $\tilde{\cdot }$ means perturbation. To ensure valid consistency regularization, the same perturbation should be applied to the geometric predictions of the original branch.

#### 3.4.2. History-Guided Label Refurbishment

**Figure 4:**
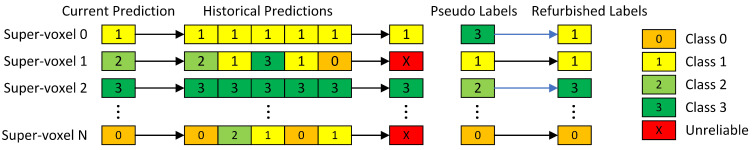
Illustration of the history-guided label refurbishment (HLR) module. We use a historical queue to store the past predictions and correct the previously generated pseudo-labels with consistently predicted classes while keeping the unreliable samples unchanged instead of directly dropping them. Compared with other methods, we take a more conservative strategy, as regularization decreases the overfitting risk.

In light of the memorization effect, in which deep networks first learn simple patterns in clean data before memorizing noise by brute force [48], the model is able to identify and correct inaccurate labels by itself during training. Specifically, the consistency of predictions is widely used as a confidence criterion [27,49,50,51]. Along this line, we consider samples with consistent historical predictions as refurbishable. The refurbishment process is illustrated in Figure 4.

Let $\Psi \left(q\right)=\left\{{\hat{y}}_{t_{1}},{\hat{y}}_{t_{2}},\cdots ,{\hat{y}}_{t_{q}}\right\}$ denote the label prediction history of a super-voxel sample, where *q* is the length of the historical queue. The frequency of the super-voxel being predicted as the *c*-th category is calculated as

(10) $$F\left(c|q\right)=\sum_{i=1}^{q}\frac{\left[{\hat{y}}_{t_{i}}=c\right]}{q},$$

where [·] is the Iverson bracket. With the frequency–probability approximation, we apply the following normalized information entropy as the consistency metric:

(11) $$H\left(q\right)=\frac{1}{Z}\sum_{c=1}^{C}-F\left(c|q\right)\log F\left(c|q\right),$$

where $Z=\sum_{c=1}^{C}-\frac{1}{C}\log\left(\frac{1}{C}\right)=\log\left(C\right)$ is the normalization term representing the maximum entropy. A smaller entropy indicates more consistent predictions. To be concrete, we treat the super-voxel that satisfies H(q)≤ϵ(0≤ϵ≤1) as the refurbishable sample. The refurbished label is defined as

(12) $$y^{*}=\underset{1\leq c\leq C}{\mathrm{argmax}}F\left(c|q\right).$$

Apparently, the refurbishment will be applied after an appropriate number of warm-up epochs, which is longer than the historical queue. The refurbishable samples are relocated at each new epoch to avoid the accumulation of correction errors. Instead of dropping the remaining samples, we leave them unaffected to enable full exploration of the dataset. In addition, it is noteworthy that we do not impose any restrictions on the label noise, which makes our refurbishment robust to different noise types and different noise rates.

#### 3.4.3. Temporal Consistency Regularization

The label refurbishment in Section 3.4.2 only utilizes discrete hard labels, overlooking the rich information in the continuous soft distributions. Here, we record the exponential moving average (EMA) of historical logits to impose temporal consistency regularization [32,33].

Let $z_{e}$ be the model’s output logits at epoch *e*. After the first trivial epoch, the moving-average logits can be normally updated as

(13) $${\bar{z}}_{e}=\left(1-\alpha \right){\bar{z}}_{e-1}+\alpha z_{e},$$

where α is the weight of the current epoch. In accordance with the conventional practice, we add the temperature τ to further soften the distribution:

(14) $$p_{ic}^{\tau }=\frac{\exp(z_{ic}/\tau )}{\sum_{k=1}^{C}\exp\left(z_{ik}/\tau \right)}.$$

**Figure 5:**
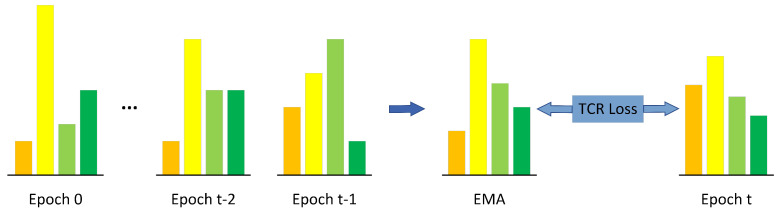
Illustration of the temporal consistency regularization (TCR) module. We record the exponential moving average of the past predicted distributions (logits), which serve as the soft targets for the current prediction.

The temporal consistency regularization term (“TCR loss” in Figure 5) is then defined as

(15) $$L_{tcr}=\frac{1}{N}\sum_{i=1}^{N}\tau^{2}D_{KL}\left({\bar{p}}_{i}^{\tau }∥p_{i}^{\tau }\right),$$

where $\bar{\cdot }$ denotes the corresponding EMA version.

With the temporal consistency regularization, the network tries to learn from itself and make comparatively stable predictions, which is important for correcting mislabeled hard samples and promoting the generalization performance.

### 3.5. Total Loss

Our SDPH can be trained in an end-to-end manner with the total loss L, which contains three parts: the basic loss $L_{basic}$, the perturbation-based consistency regularization loss $L_{pcr}$, and the temporal consistency regularization loss $L_{tcr}$.

(16) $$L=L_{basic}+L_{pcr}+L_{tcr},$$

where $L_{basic}$ is given in Equation (2), $L_{pcr}$ is given in Equation (7), and $L_{tcr}$ is given in Equation (15). As we mentioned before, the label refurbishment needs a warm-up stage in which the noisy labels are unchanged in $L_{basic}$. That is why we call it the “noisy loss” in Figure 2. After the warm-up stage, $L_{basic}$ is referred to as the “clean loss”, since the labels have been cleaned.

## 4. Experiments

### 4.1. Experimental Settings

#### 4.1.1. Dataset

**Figure 6:**
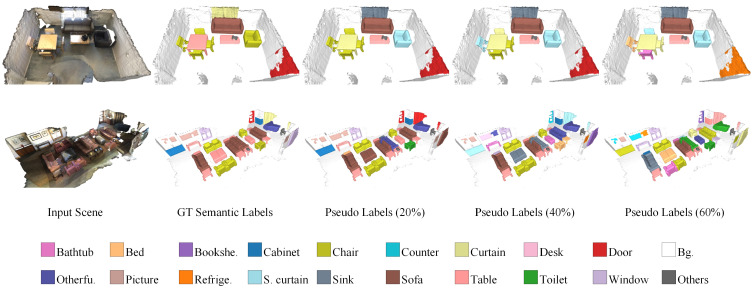
Visualization of different noise rates affecting the semantic labels. From left to right are the input scene, the ground-truth semantics, and the pseudo-labels of noise rates of 20%, 40%, and 60%. The higher the noise rate, the more chaotic the semantics.

We conducted experiments on the widely used ScanNet-v2 [15] dataset. This challenging large-scale indoor point cloud dataset consists of 1201 training scenes, 312 validation scenes, and 100 hidden testing scenes. Each scene of the training and validation sets is richly annotated with point-level semantic-instance labels that are used in the densely supervised methods. However, we created axis-aligned bounding boxes from the point-level annotations to validate our weakly supervised learning framework. To simulate inaccurate annotations, we artificially injected symmetric noise into the training set. Specifically, the semantic labels of the corrupted instance boxes were changed to other labels with equal probability. We used the noise rate, i.e., the probability of each box being mislabeled, to represent the severity of inaccurate supervision. The effects of different noise rates are visualized in Figure 6.

#### 4.1.2. Evaluation Metrics

As with existing methods, we used the mean average precision over 18 foreground object categories as our evaluation metric. To be specific, $AP_{25}$ and $AP_{50}$ denote the scores with IoU thresholds set to 0.25 and 0.5, respectively. In addition, we also report the AP, which averages scores with thresholds varying from 0.5 to 0.95, with a step size of 0.05.

#### 4.1.3. Implementation Details

All experiments were performed on a PC with two NVIDIA GeForce RTX 3090 Ti GPUs and an Intel Core i7-12700K CPU. We used two GPUs for distributed training and one for inference. The main software configuration included Python 3.8.13, Pytorch 1.10.2, CUDA 11.3, and MinkowskiEngine 0.5.4. Following the pioneering work of Box2Mask [20], we adopted a six-layer UNet-like sparse convolutional network as our backbone, and the multi-head MLPs were implemented with three layers and 96 hidden units. We set the voxel size to 0.02 m. For history-guided label refurbishment, we set the number of warm-up epochs, the length of the historical queue, and the threshold ϵ to 40, 10, and 0.001, respectively. For temporal consistency regularization, the temperature τ and the EMA coefficient α were empirically set to 3 and 0.9. We trained our network from scratch with a batch size of 4 for 200 epochs in total while using the Adam optimizer with an initial learning rate of 0.001. A cosine annealing scheduler was applied after 100 epochs.

### 4.2. Instance Segmentation Results

**Figure 7:**
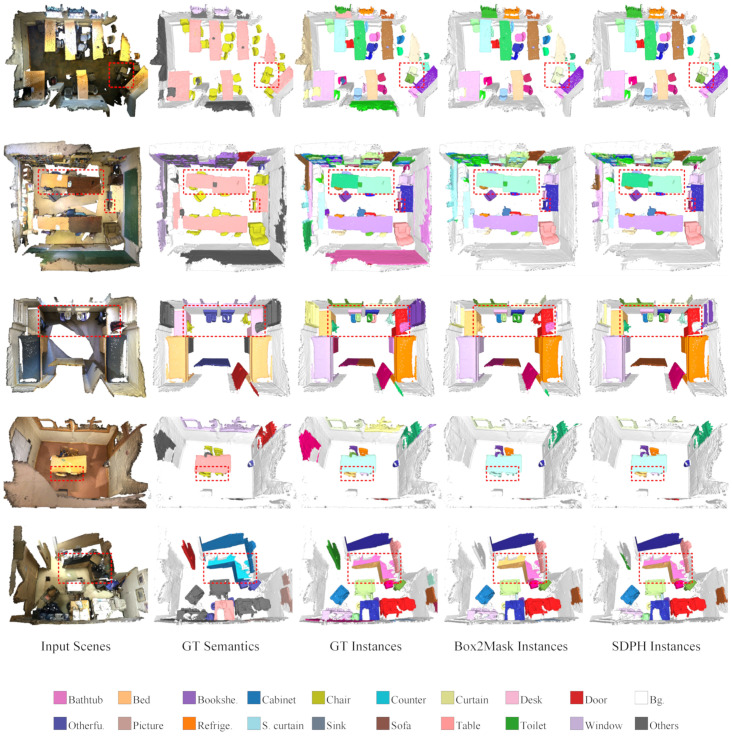
Qualitative comparison at a noise rate of 40% on ScanNet-v2. The legend is employed to distinguish among different semantic meanings, while the individual instances are randomly colored. The key differences are marked out with red dashed rectangles.

**Figure 8:**
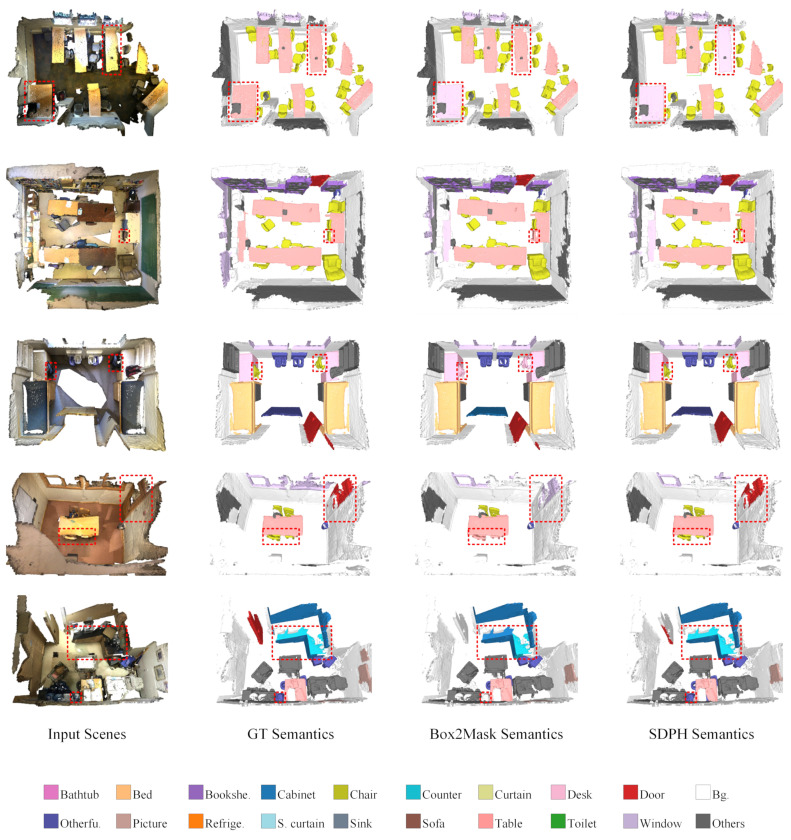
Qualitative comparison at a noise rate of 40% on ScanNet-v2. The legend is employed to distinguish among different semantic meanings, and the key differences are marked out with red dashed rectangles.

First of all, we conducted comparative experiments with different noise rates to demonstrate the effectiveness of our noise-tolerant learning framework, SDPH. As listed in Table 1, our SDPH achieved consistently better performance than that of Box2Mask (the baseline) under all of the noise rate settings with respect to all of the evaluation metrics. From the overall trend, we observed that higher noise rates were related to larger improvements. When the noise rate is set to 40%, our SDPH still outperformed noise-free Box2Mask in terms of AP. The performance was comparable or even better in terms of $AP_{25}$ and $AP_{50}$ when the noise rate was 20%. These results demonstrate our method’s robustness to label noise. Qualitative comparisons of instance and semantic segmentation are shown in Figure 7 and Figure 8, respectively. When training with a noise rate of 40%, our SDPH predicted the semantics more accurately than Box2Mask did, which usually led to better instance segmentation performance.

Even though our method was designed especially for learning with label noise, we acquired a little performance gain in the “noise-free” setting. The possible reasons are two-fold. Firstly, Box2Mask trained the network with associated super-voxel-level pseudo-labels that were not inaccurate. Hence, the label refurbishment worked even without additional noise injection. Secondly, our SDPH benefited from the regularization terms that distilled knowledge from the data and the model itself.

**Figure 9:**
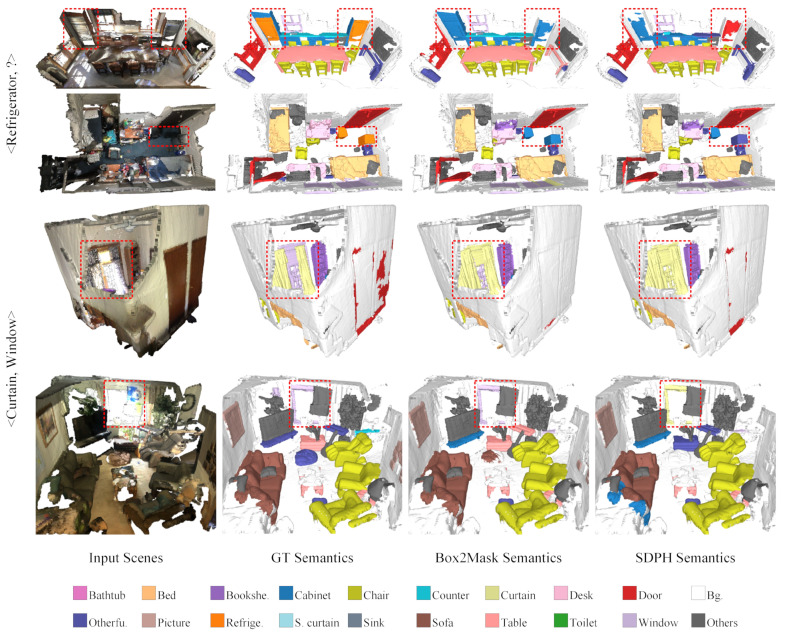
Bad cases on ScanNet-v2 in the noise-free setting. The first two rows show that refrigerators could be misclassified as cabinets, doors, and other furniture. We use “?” to represent this complicated situation. The last two rows show that windows could be misclassified as curtains, which lowered both categories’ performance. The legend is employed to distinguish among different semantic meanings, and the key differences are marked out with red dashed rectangles.

In Table 2, we provide a detailed comparison with state-of-the-art methods that do not explicitly consider label noise. It can be seen that our method performed well in the noise-free setting, which demonstrated the effectiveness of our SDPH. However, instead of attaining consistent performance boosts over different categories, there were some significant declines and increases, especially between SDPH and 3D-MPA [9]. This was probably because 3D-MPA and SDPH adopted different supervision types and instance segmentation routines. The former is a proposal-based method with point-level supervision, while our SDPH is proposal-free and uses the more challenging box-level supervision. As proposal-free methods, PointGroup [38], Box2Mask [20], and SDPH exhibited similar trends when compared with 3D-MPA. For example, their performance greatly declined for refrigerators and shower curtains, and it increases for chairs, desks, sinks, sofas, and other furniture. As shown in Figure 9, the refrigerators had various shapes and sizes and were sometimes surrounded by cabinets. Moreover, curtains were usually beside windows. Even in the case of full point-level supervision—let alone weak box-level supervision—it was difficult to segment them clearly. Furthermore, compared with chairs and desks, there were fewer instances of these categories, which could lead to SDPH’s false refurbishment and lower performance.

### 4.3. Ablation Study

We analyzed the contribution of each component in our learning framework, including perturbation-based consistency regularization (PCR), history-based label refurbishment (HLR), and temporal consistency regularization (TCR). It should be noted that the models in the ablation study were all trained with a noise rate of 40%. The complete ablation results are shown in Table 3. We found that every single component was able to improve the performance by itself. In particular, TCR alone obtained 2.6, 3.4, and 2.0 percent improvements in terms of AP, $AP_{50}$, and $AP_{25}$, respectively. The performance could be further boosted through their combination, and the largest increases in AP, $AP_{50}$, and $AP_{25}$ reached 5.2, 5.3, and 3.2 by combining all three components. This thorough ablation study demonstrated that each module plays an important role in our framework.

### 4.4. Analysis of Label Refurbishment

**Figure 10:**
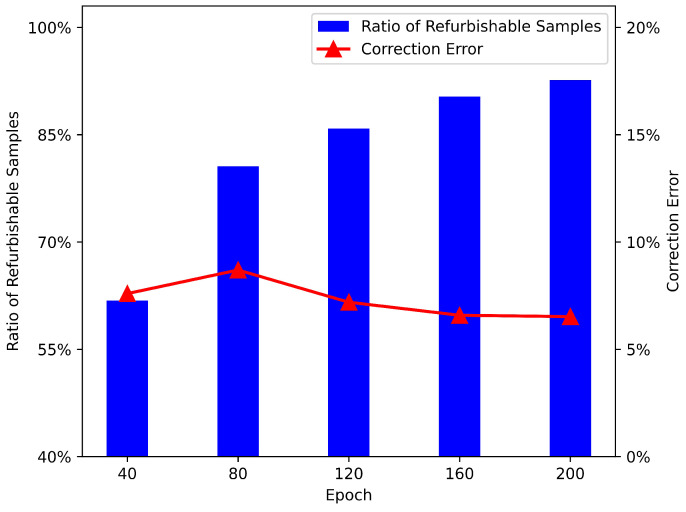
Trend of statistics in history-guided label refurbishment.

To demonstrate the process of label refurbishment, we further recorded two related statistics, as shown in Figure 10. The first was the ratio of refurbishable super-voxel samples, which was defined as

(17) $$\eta =\frac{number\;of\;refurbishable\;samples}{number\;of\;total\;samples}.$$

The second was the correction error, which could be computed as

(18) $$\delta =\frac{number\;of\;mistakenly\;corrected\;samples}{number\;of\;refurbishable\;samples}.$$

Note that both statistics took the entire training set into account. We set the noise rate to 40%.

**Figure 11:**
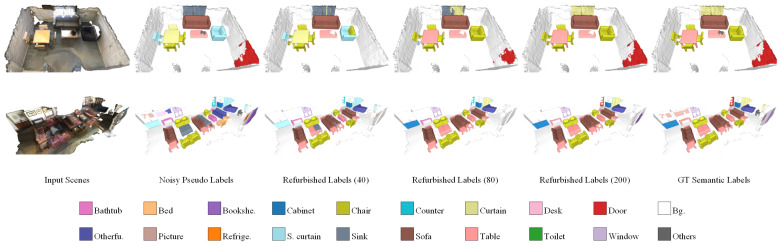
Qualitative demonstration of history-guided label refurbishment. From left to right are the input point clouds, the corresponding noisy pseudo-labels, the refurbished labels in epochs 40, 80, and 200, and the ground-truth semantic labels.

The ratio of refurbishable super-voxel samples gradually increased from 61.8% to 92.6%, finally covering the majority of the whole training set. Moreover, the correction error stayed relatively low throughout the training process because we adopted a conservative refurbishment strategy. On the one hand, the refurbishable threshold was quite strict to reduce false correction. On the other hand, we kept the unrefurbishable samples instead of dropping them, which lowered the risk of error accumulation. Therefore, the label quality was steadily improved as the training proceeded, as shown in Figure 11. However, we observed that it was easier to correct the labels of isolated objects with clear boundaries, such as chairs, sofas, and tables. On the contrary, flat objects that were often attached to walls, such as pictures and curtains, were harder to distinguish from the background.

### 4.5. Complexity Analysis

Apart from the mean average precision, we also compared the time costs to give a full picture of the performance. As shown in Table 4, the inference time of our SDPH was comparable to that of the state-of-the-art weakly supervised method Box2Mask [20], though SDPH required a longer time for training. In fact, our approach mainly focused on the design of loss functions that only affected the training cost. Without extra network parameters, the majority of the additional cost came from perturbation-based consistency regularization (PCR), since it constructed a perturbed network branch. PCR did not affect the inference time, as only the main branch was used in inference.

## 5. Conclusions

In this work, we proposed a novel self-distillation architecture for weakly supervised point cloud instance segmentation with inaccurate bounding boxes as annotations. We employed consistency regularization based on data perturbation and historical records to prevent the network from overfitting noisy labels. Moreover, the noisy labels were refurbished according to the predictions’ temporal consistency without knowing the noise rate. An extensive ablation study and analysis verified the importance of each module in SDPH. Our method achieved comparable performance to that of fully supervised methods, and it outperformed recent weakly supervised methods by at least 1.2 percentage points in terms of $AP_{25}$, which demonstrated the effectiveness and robustness of our framework.

In the future, we plan to extend the noise types to asymmetric semantic noise and geometric coordinate noise, which may require a new confidence criterion. In addition, inspired by the mutual promotion between semantic segmentation and instance segmentation, semantic classification and geometric regression could be associated through smoothness regularization to reduce discontinuity and messy “over-segmentation”.

## Figures and Tables

**Table 1:** Quantitative comparison of different noise rates on ScanNet-v2.

Method	Metric	0%	10%	20%	30%	40%	50%	60%
Box2Mask [20]	AP	39.1	37.5	36.3	36.3	35.2	33.6	32.0
	$AP_{50}$	59.7	57.5	55.8	55.4	53.3	50.4	46.7
	$AP_{25}$	71.8	69.8	68.8	67.3	65.8	62.6	58.2
SDPH	AP	40.1	41.2	40.8	40.0	40.4	37.6	36.5
	$AP_{50}$	60.4	60.4	60.3	58.7	58.6	55.1	52.5
	$AP_{25}$	73.0	72.1	71.7	70.7	69.0	65.4	61.9
Improvements	AP	1.0	3.7	4.5	3.7	5.2	4.0	4.5
	$AP_{50}$	0.7	2.9	4.5	3.3	5.3	4.7	5.8
	$AP_{25}$	1.2	2.3	2.9	3.4	3.2	2.8	3.7

**Table 2:** Quantitative comparison with state-of-the-art methods on ScanNet-v2. The highest performance in each column is marked in bold.

Setting	Method	${AP}_{25}$	Bathtub	Bed	Bookshe.	Cabinet	Chair	Counter	Curtain	Desk	Door	Otherfu.	Picture	Refrige.	S. Curtain	Sink	Sofa	Table	Toilet	Window
Full	SegCluster [35]	13.4	16.4	13.5	11.7	11.8	18.9	13.7	12.4	12.2	11.1	12.0	0.0	11.2	18.0	18.9	14.6	13.8	19.5	11.5
	SGPN [11]	22.2	0.0	31.5	13.6	20.7	31.6	17.4	22.2	14.1	16.6	18.6	0.0	0.0	0.0	52.4	40.6	31.9	72.9	15.3
	3D-SIS [35]	35.7	57.6	66.3	16.9	32.0	65.3	22.1	22.6	35.1	26.7	21.1	0.0	28.6	37.2	39.6	56.4	29.4	74.9	10.1
	MTML [52]	55.4	79.4	80.6	45.3	34.6	87.7	9.7	54.2	49.9	45.8	33.5	19.8	44.1	74.9	44.5	80.3	67.4	98.0	47.2
	PointGroup [38]	71.3	86.5	79.5	74.4	67.3	92.5	**64.8**	61.6	74.1	54.8	65.4	**48.2**	38.3	71.1	82.8	85.1	74.2	**100**	**63.6**
	3D-MPA [9]	72.4	**90.3**	83.4	**78.3**	**69.9**	87.6	62.5	**66.0**	69.2	56.6	48.6	48.0	**61.4**	**93.1**	75.2	76.1	74.8	99.2	62.2
Weak	SPIB [22]	61.4	87.4	**86.8**	48.8	45.4	89.0	49.6	47.8	52.3	49.2	45.5	9.9	48.3	82.6	63.2	**88.1**	66.2	95.9	41.9
	Box2Mask [20]	71.8	87.1	83.8	68.2	59.5	94.5	58.5	65.1	78.6	59.8	**67.1**	45.6	46.9	77.4	79.5	87.0	75.5	96.9	61.4
	SDPH	**73.0**	87.1	82.6	73.6	62.1	**95.2**	63.0	61.5	**85.5**	**61.1**	63.1	43.5	46.7	82.0	**85.4**	86.3	**78.2**	98.3	59.3

**Table 3:** Ablation study on ScanNet-v2. The highest performance in each column is marked in bold.

PCR	HLR	TCR	AP	${AP}_{50}$	${AP}_{25}$
			35.2	53.3	65.8
√			37.1	53.7	65.1
	√		37.6	55.4	66.6
		√	37.8	56.7	67.8
√	√		39.5	58.1	67.9
√		√	37.1	54.8	65.6
	√	√	39.5	57.4	68.8
√	√	√	**40.4**	**58.6**	**69.0**

**Table 4:** Comparison of the average computation time in milliseconds per scan on ScanNet-v2. The running time was measured in the same environment. Note that a post-processing step was implemented to cluster points into instances in inference.

Method	Training Time (ms)	Inference Time (ms)
Box2Mask [20]	444	1044
SDPH	722	1026

## Data Availability

Publicly available datasets were analyzed in this study. This data was accessed at http://www.scan-net.org/ on 27 August 2022.
